# Feasibility of a registry for standardized assessment of long-term and late-onset health events in survivors of childhood and adolescent cancer

**DOI:** 10.1038/s41598-022-18962-7

**Published:** 2022-08-26

**Authors:** Maria Otth, Daniel Drozdov, Katrin Scheinemann

**Affiliations:** 1 Division of Oncology-Haematology, Department of Pediatrics, Kantonsspital Aarau, Aarau, Switzerland; 2 Department of Oncology, Haematology, Immunology, Stem Cell Transplantation and Somatic Gene Therapy, University Children’s Hospital Zurich, Zurich, Switzerland; 3 Department of Health Sciences and Medicine, University of Lucerne, Lucerne, Switzerland; 4 Department of Pediatrics, McMaster Children’s Hospital, Hamilton, ON Canada; 5 McMaster University, Hamilton, ON Canada

**Keywords:** Cancer epidemiology, Paediatric cancer, Disease prevention

## Abstract

Childhood and adolescent cancer survivors are at risk for chronic medical conditions. Longitudinal studies help to understand their development and course. We hypothesize that collecting follow-up data according to the modified CTCAE criteria and embedded in regular care, is feasible and results in a rich database. We recruited 50 Swiss survivors treated at our institution between 1992 and 2015, who completed their treatment and are still alive. Information on cancer diagnosis, treatment, and medical conditions from follow-up visits, graded according to the modified CTCAE criteria, were added in the database. We described the cohort, assessed the prevalence of medical conditions at the most recent visits and the time needed for data entry. Survivors had a median age of 10 years at diagnosis with 16 years of follow-up. 94% of survivors suffered from at least one medical condition. We registered 25 grade 3 or 4 conditions in 18 survivors. The time needed for data entry at enrollment was < 60 min in most survivors and much less for follow-up visits. Standardized assessment of medical conditions is feasible during regular clinical care. The database provides longitudinal real-time data to be used for clinical care, survivor education and research.

## Introduction

Around 400 children and adolescents younger than 20 years of age are diagnosed with cancer annually in Switzerland^1^. The 10 years survival rate currently exceeds 87% for all types of cancer combined and results in increasing numbers of long-term childhood cancer survivors (CCSs)^1,2^. Recent study results confirm that the majority of CCSs, treated for childhood and adolescent cancer between 1960 and 2000s, are diagnosed with chronic medical conditions, so called late effects, due to the cancer itself or its treatment^3–5^. Every organ system, including the respiratory, cardiovascular, sensorineural and endocrine system, as well as skin, central nervous system, immune system and others, can potentially be affected by chronic medical conditions. Two third (62.3%) of American CCSs diagnosed between 1970 and 1986 reported at least one chronic medical condition and 27.5% were defined as grade 3 or 4 conditions^4^. The 10-years survivors included in the St. Jude Lifetime Cohort Study (SJLIFE), diagnosed between 1961 and 2004, show a cumulative incidence of medical conditions at age 50 years of 99.9% for grade 1–5 and 96% for grade 3–5 conditions^5^. These results highlight that long-term follow-up care through childhood, adolescence and into adulthood is of utmost importance for CCSs.

The long-term follow-up recommendations from the Children’s Oncology Group (COG) and the International Late Effects of Childhood Cancer Guideline Harmonization Group (IGHG) are useful tools to estimate the individual risk of each CCS to develop chronic medical conditions and to formulate screening recommendations^6,7^. The screening recommendations together with the treatment overview can be summarized individually for each CCS in a survivorship care plan^8^. The survivorship care plan serves as a guidance for CCSs and treating physicians and should be updated in case of new evidence.

To describe medical conditions within single CCSs longitudinally but also between different CCSs it is important that the outcomes are assessed in a standardized way. The Common Terminology Criteria for Adverse Events (CTCAE) is a widely used descriptive terminology designed to report and grade medical conditions of each organ system^9^. Hudson et al.^10^ adapted the CTCAE criteria to meet the special requirements of CCSs. We therefore incorporated these modified CTCAE criteria in the “Young Survivor at KSA (Kantonsspital Aarau)” registry^11^. The "Young Survivors at KSA" registry collects information on treatment exposures that are known to or might cause chronic medical conditions in CCSs. It additionally gathers information retro- and prospectively on medical conditions of organ systems at risk based on the COG and IGHG guidelines in a standardized way by using the modified CTCAE criteria. The registry allows to assess the frequency, severity, and longitudinal changes of these medical conditions over time^11^. The primary purpose of this study is to assess the feasibility of performing the data collection during regular follow-up care and to describe the first 50 survivors randomly recruited in the registry.

## Methods and materials

### Study population

We randomly recruited 50 CCSs, defined as former childhood cancer patients who were diagnosed before 18 years of age and had entered follow-up care. They had therefore completed the cancer treatment at recruitment. The CCSs were still alive, were treated between 1992 and 2015 and still in follow-up care in the Division of Oncology-Haematology, Department of Pediatrics, Kantonsspital Aarau. In this division, CCSs who become young adults are transitioned into adult-focussed follow-up care through combined consultations with pediatric and adult physicians^12^.

### Survivorship care plan

Either during a long-term follow-up visit in the pediatric setting or latest at the transition visit, each CCS receives a survivorship care plan with detailed information about the diagnosis, treatment received, risks to potentially develop medical conditions, and recommended health care screening in the future. These plans are part of routine clinical care at the Division of Oncology-Haematology in the Department of Pediatrics, Kantonsspital Aarau. The information on diagnosis and treatment each CCS received is needed for the survivorship care plans. For treatment exposure, detailed information on chemotherapeutic agents, including cumulative doses, radiation doses, radiation fields, surgeries and hematopoietic stem cell transplantation are collected. This information is extracted from the electronic patient records, which were systematically introduced in 2016 in all pediatric and adult departments of the Kantonsspital Aarau. Most of the examinations performed before 2016 were scanned into these electronic records. This enables the easy extraction of information from consultation letters, laboratory results, radiology or nuclear medicine reports, lung function tests, echocardiography, audiometry, visual testing and others. For older data, there is still access to the paper-based records, which are kept beyond the maximal storage time of 20 years required by the Swiss law.

### Organ systems at risk

For each CCS, we defined the individual organ systems at risk to develop medical conditions according to the COG and IGHG guidelines^6,7^. The treatment received defines the organ systems at risk. For example, a CCS exposed to anthracyclines is at risk for cardiac conditions and the frequency of cardiac assessment depends on the cumulative dose and additional risk factors like radiotherapy.

### Data entry and database

The detailed information on treatment exposure gathered in the survivorship care plan are used for the "Young Survivors at KSA" registry. These data are entered manually in the registry-specific electronic database (REDCap)^13,14^ (details on the database is available in the study protocol^11^). Based on the modified CTCAE criteria, medical information on 15 organ systems: auditory, cardiovascular, endocrine, gastrointestinal, hematological, hepatobiliary, immunological, infectious, pulmonary, musculoskeletal, neurological, neurocognitive, renal/urinary tract, reproductive/genital tract and ocular/visual are additionally entered in the database^10^. The information on organ systems at risk is extracted in annual intervals from the medical records at the time of the annual visit. For the information on organ systems at risk, we assumed that the retrospective data collection might result in missing data in some CCSs. For these situations, we have foreseen to leave the corresponding organ systems blank, but to note in the database that the CCS is at risk. Data extraction and entry was performed manually by one author and spot check verified by another author. If an incorrect data entry was noticed, it was corrected and all data from the respective year were checked (details on methods is available in the study protocol^11^).

### Ethical considerations and statistical analysis

The “Young Survivors at KSA” registry was approved by the cantonal ethics committee EKNZ (AO_2020-00012), is registered on ClinicalTrials.gov (NCT04811794), was conducted in accordance with the Declaration of Helsinki and the principles of Good Clinical Practice, and written informed consent was obtained from all participants^11,15,16^. For this feasibility study, only the information on organ function from the most recent follow-up visit was analyzed. It was assumed that most CCSs have been diagnosed with leukemia and lymphoma and therefore the diagnoses were stratified into “leukemia and lymphoma” and “others”. This decision was taken based on the knowledge that leukemia and lymphoma belong to the three most frequent cancer types in children and adolescents, have among the highest survival rates and belong to the largest groups of long-term CCSs in other cohorts^17–19^. The time needed to complete the first entry into the database was assessed for each CCS as either up to 60 min, 60–90 min or 90–120 min. STATA (StataCorp. LCC, Version 17. College Station, TX) was used as statistical software to describe the study population and medical conditions descriptively and to present the data on time needed to perform the data entry. Details on the registry can be found in the respective study protocol^11^.

## Results

**Figure 1:**
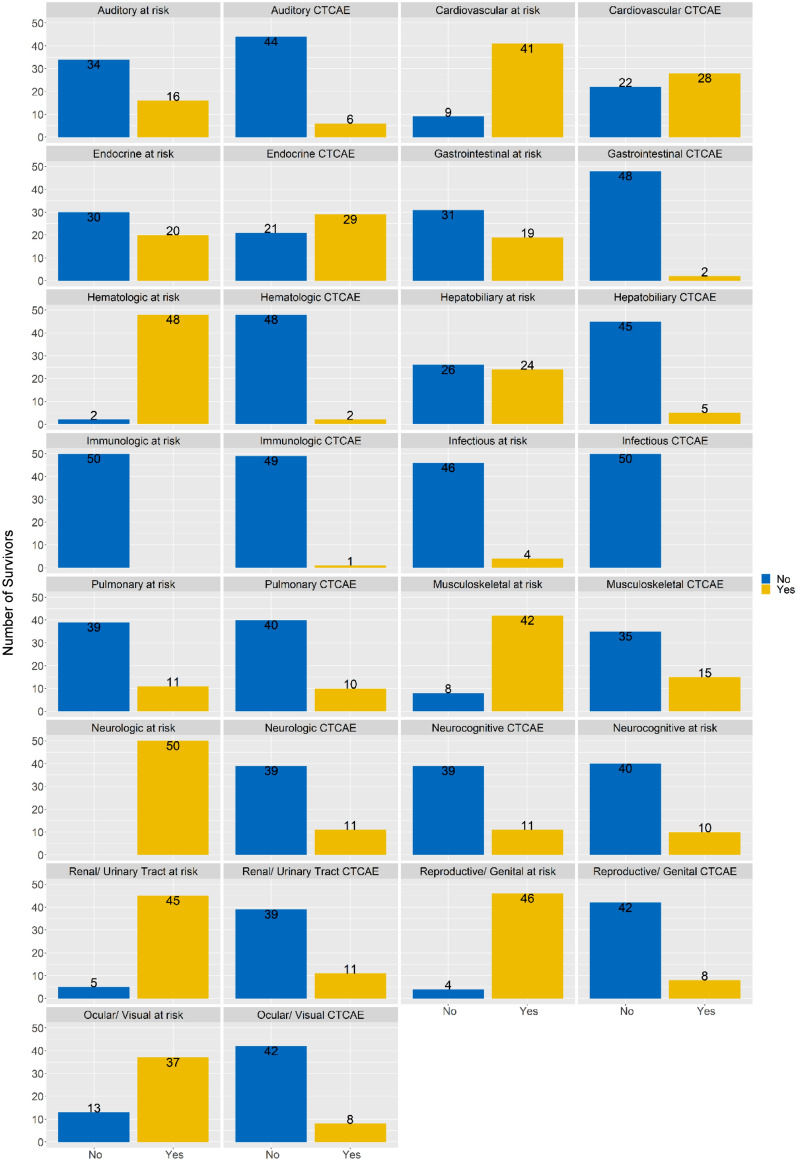
Number of childhood cancer survivors at risk (column 1 and 3) and diagnosed according to CTCAE criteria (column 2 and 4) with a medical condition at their last follow-up visit. X-axis: yellow = at risk or diagnoses with a condition (“yes”), blue = not at risk or not diagnosed with a condition (“no”). Y-axis: number of survivors. Being at risk defined by COG survivorship guidelines.

Half of the CCSs were female (n = 25) with a median age of 10 years (IQR 3.60–12.56) at diagnosis with 16 years (IQR 12.64–19.48) of follow-up. One fourth of CCSs were diagnosed with leukemia (26%) or lymphoma (24%) each (Table 1). In most CCSs (n = 47), at least one medical condition of any grade was recorded during the most recent follow-up visit. One quarter (28%) of CCSs had one medical condition only, with a maximum of 18 conditions in one CCS. Most medical conditions were grade 1 or 2 (87%). Based on the COG guidelines, all CCSs were at risk for neurological conditions and in 22% of CCSs (n = 11) such a condition was registered (Fig. 1, Supplemental Table 1). More than 90% of all CCSs were at risk for hematologic, renal/urinary tract, or reproductive conditions (Fig. 1). At their last visit, 2 CCSs suffered from a hematological condition and 11 from a renal/urinary tract condition, including 10 CCSs with a chronic kidney disease grade 1 and one CCS with a grade 2 disease. Additional 8 CCSs suffered from a condition affecting the reproductive system (Supplemental Table 1). On the other side of the frequency spectrum, no CCS was at risk for immunological conditions, but one presented with immunoglobulin A deficiency. Less than 10% of CCSs were at risk for infectious conditions and none presented with outcome findings. Endocrine (n = 29) and cardiovascular conditions (n = 28) were detected most frequently during the last visit, including CCSs at risk and not at risk. The endocrine conditions mainly included obesity grade 2 (n = 16), obesity grade 3 (n = 6), and hypothyroidism grade 2 (n = 9), with these conditions not being mutually exclusive (Supplementary Table 1). The cardiovascular conditions consisted of arterial hypertension grade 1 (n = 22), arterial hypertension grade 2 (n = 2), heart valve disorder (n = 2), high total cholesterol (n = 6), and hypertriglyceridemia (n = 7). Detailed information on CTCAE events recorded in all 15 disease categories are summarized in Supplementary Table 1.

**Table 1:** Clinical characteristics of participating childhood cancer survivors (N = 50).

	**n (%)**
**Gender**	
Female	25 (50)
**Cancer type**	
Leukamia	13 (26)
Lymphoma	12 (24)
Central nervous system neoplasms	7 (14)
Renal tumors	4 (8)
Liver tumors	1 (2)
Malignant bone tumors	3 (6)
Soft tissue sarcomas	5 (10)
Germ cell tumors	5 (10)
**Relapse**	
Yes	4 (8)
**Secondary malignancy**^**a**^	
Yes	3 (6)
**Age at diagnosis [years] (median [IQR])**	10.1 [3.6–12.6]
0–5 years	16 (32)
6–10 years	9 (18)
11–15 years	21 (42)
16–18 years	4 (8)
**Follow up time since diagnosis [years] (median [IQR])**	16.1 [12.6–19.5]
5–10 years	7 (14)
11–15 years	15 (30)
16–20 years	16 (32)
21–25 years	9 (18)
26–30 years	3 (6)

^a^Secondary malignancies: papillary thyroid carcinoma (n = 1), pilocytic astrocytoma in a patient with neurofibromatosis type 1 (n = 1), basal cell carcinoma (n = 1); IQR, interquartile range.

The time needed to complete the first data entry in the electronic database, including information on patient history, diagnosis, treatment, and medical data of all follow-up visits that took place between 2016 and 2021, was up to 60 min in 40 CCSs and between 60 and 90 min in 10 CCSs. The time needed to complete the prospective follow-up visits was around 15 min each. The data entry of all CCSs was feasible during regular working time. The follow-up visits were entered directly following the outpatient visits.

## Discussion

In 94% of the 50 included CCSs at least one medical condition was detected during the last follow-up visit, at a median of 16 years from diagnosis. Most of the condition were grade 1 and 2 according to the modified CTCAE criteria. For most CCSs the first and very comprehensive data entry into the database took less than 1 hour with much less time needed for the follow-up entries.

The present results are comparable to those from the SJLIFE cohort, where Bhakta et al.^5^ assessed the cumulative incidence of medical conditions at age 50 years in 3010 CCSs, diagnosed between 1971 and 2004. In both cohorts, leukaemia was the most frequent diagnosis (this study 26%, SJLIFE 34%), followed by lymphoma (this study 24%, SJLIFE 20%) and central nervous system neoplasms (this study 14%, SJLIFE 12%)^5^. In both studies, the modified CTCAE criteria were used to grade the medical conditions, but the outcomes were reported differently. This study used the prevalence at last visit, and the cumulative incidence at age 50 years was used in the SJLIFE study. However, in both studies, endocrine (58% vs 92% SJLIFE) and cardiovascular (56% vs 93% SJLIFE) conditions were reported most frequently. The difference in follow-up times with 16 years in the present study and attained age of 50 years in the SJLIFE study might explain why a smaller proportion of CCSs suffered from the conditions in the present study. Howell et al.^18^ described the prevalence of medical conditions in 5223 CCSs according to the modified CTCAE criteria at baseline assessment into SJLIFE. Focussing on CTCAE grade 3 and 4 only, endocrine conditions were reported in 16.6% of SJLIFE participants and 16% in the present cohort, and cardiovascular conditions in 7.2% in SJLIFE and 2% in the present cohort. One cohort study from the Netherlands invited CCSs diagnosed between 1963 and 2001 for a medical assessment in a Late Effects Clinic. Among 6165 included CCSs, 261 (4.2%) developed at least one secondary cancer^20^. In our cohort, 3 out of 50 CCSs (6%) developed secondary cancers. This proportion was 3.7% in the 5223 CCSs from the SJLIFE cohort^18^. The proportion was highest in the present cohort, which might be explained by the relatively small sample size, where each single CCS gives more weight. This hypothesis is further supported by the observation that the median follow-up time was longer in the Dutch cohort than in the present cohort (20.7 years versus 16.1 years) and CCSs were diagnosed in earlier decades. Both observations would lead to the assumption that the proportion should be higher in the Dutch cohort. However, the source data differ, and the Dutch study mainly used linkage with the cancer and pathology registries to identify secondary cancer. Gathering information from medical records directly might result in higher proportions. A further explanation for the different proportions of secondary cancers might be differences in defining secondary cancer, as this was not described in the Dutch and the SJLIFE cohort. Secondary cancer was defined in this study as such, that the histology was not identical to the histology of the primary cancer.

It was not possible to compare the time and effort needed to complete the study database within clinical practice to other longitudinal cohort studies, as this information does not exist. The experience from this feasibility study shows that it is possible to integrate the completion of such a database during regular clinical practice, meaning to enter the data directly following the consultation. With each CCS entered in the database the authors became more familiar with the structure and faster with the data entry. The authors therefore recommend that the data entry should be performed by a dedicated team or selected team members who preferably also perform the clinical visit and are experienced in long-term follow-up care.

The design of the underlying registry allows the longitudinal collection of medical data of CCSs still in follow-up care, providing real-time data with annual data points from risk-adapted screening. From a research perspective, this allows the timely analysis and evaluation of medical conditions in CCSs. This also includes children and adolescents who received newer treatment modalities, for example checkpoint inhibitors, immune modulating drugs or newer radiation techniques. From a clinicians’ perspective, monitoring individual organ function trajectories may help in patient education, for example through plotting individual CTCAE grades over time. The design would also give the possibility to link self-reported outcomes, collected separately, with the objective parameters collected within the modified CTCAE criteria. This is currently not part of the registry. The self-reported outcomes could be collected by using the pediatric, caregiver or adult version of the Patient-Reported-Outcome-CTCAE (PRO-CTCAE)^21^.

## Limitations and strengths

The comparison of these results with other studies is limited by the small sample size. However, the sample size was large enough to assess the feasibility of completing a registry database within clinical routine. The data entries of all 50 CCSs could be performed during routine clinical care and did not need extra time. As this was feasible for 50 randomly chosen CCSs it seems to be feasible for any CCS. Adding the information of each follow-up visit directly into the database is the cornerstone to prospectively feed such a database. Further, there are currently no data available that validated the use of modified CTCAE criteria in clinical settings and outside of research. In our opinion, using the modified CTCAE criteria for example to illustrate and explain the longitudinal change of a cardiac parameter to a CCS by using the categories given by the modified CTCAE criteria could add clinical value and influence therapy. This approach can be used for all organ systems where modified CTCAE criteria are available. Incompleteness of medical records and inaccuracy of collected data might be a further limitation, especially for data collected retrospectively. However, for most organ systems the source data (e.g. laboratory values, reports from echocardiograms, measured height and weight) were available electronically or in paper format, which reduced the risk for inaccuracy. Using internationally recognized long-term follow-up care guidelines to define CCSs at risk and a standardized grading system to categorize the severity of medical conditions is a key strength. Data entry was performed by one author only, spot-checked by a second author, which caused consistency.

## Conclusion

This feasibility study confirms that detailed and longitudinal data collection including patient history on diagnosis, treatment exposure, and medical conditions graded according to the modified CTCAE criteria is feasible within the regular clinical practice. The process is significantly simplified when the patient history is already summarised in a survivorship care plan and a transition into adult-care is well-established whereby the information can be collected in the pediatric and adult setting. The implementation of such a database and standardized assessment of medical conditions in other clinics would allow collaborative research with large datasets, which are needed in the field of pediatric oncology and especially survivorship care. Results of this constantly growing and real-time database might increase our understanding on medical conditions and contribute to improvement in long-term follow-up care of CCSs.

## Supplementary Information

Supplementary Table 1.

## Data Availability

The data that support the findings of this study are available on request from the corresponding author. The data are not publicly available due to privacy or ethical restrictions.

## Abbreviations

CCS(s): Childhood cancer survivor(s)
COG: Children’s Oncology Group
CTCAE: Common Terminology Criteria for Adverse Events
IGHG: International Late Effects of Childhood Cancer Guideline Harmonization Group
KSA: Kantonsspital Aarau
SJLIFE: St. Jude Lifetime Cohort Study
