# Supplementary material for: Feasibility of a registry for standardized assessment of long-term and late-onset health events in survivors of childhood and adolescent cancer
Source: Sci Rep. 2022 Aug 26;12:14617. doi: 10.1038/s41598-022-18962-7 (PMC9418307; doi:10.1038/s41598-022-18962-7)
Supplement: Supplementary file 1 — Supplementary Table 1. [file 41598_2022_18962_MOESM1_ESM.pdf]

Supplementary material

Feasibility of a Registry for Standardized Assessment of Long-Term and Late-Onset Health Events in Survivors of Childhood and Adolescent Cancer

Maria Otth, Daniel Drozdov, Katrin Scheinemann

Corresponding Author:
Maria Otth, MD
Kantonsspital Aarau
Tellstrasse 25
Aarau, 5001
Switzerland
Phone: 41 62 838 41 41
Email: maria.otth@ksa.ch

Supplementary Table 1: Frequency of medical conditions according to modified CTCAE criteria. Stratified by survivors diagnosed with leukemia/ lymphoma and other diagnoses; being at risk defined by COG survivorship guidelines; more than one condition possible per survivor.

		Grade	Leukemia and Lymphoma	Other diagnosis
n			25	25
**Hematologic**				
**Survivors at risk**	**Yes**		**25**	**23**
**Hematologic outcomes (CTCAE)**	**Yes**		**1 (1 at risk)**	**1 (0 at risk)**
Anemia		1	1	0
**Neurologic**				
**Survivors at risk**	**Yes**		**25**	**25**
**Neurologic outcomes (CTCAE)**	**Yes**		**1 (1 at risk)**	**10 (10 at risk)**
Cerebellar dysfunction		1	0	1
		2	0	2
Cranial nerve disorder		1	0	2
		2	0	3
Dysarthria		2	0	1
Movement disorders		1	0	1
Nerve root disorder		**3**	**0**	**1**
Peripheral motor neuropathy		2	0	1
Peripheral sensory neuropathy		1	0	1
		2	0	1
Seizures		2	1	0
		**3**	**0**	**1**
**Renal/ Urinary Tract**				
**Survivors at risk**	**Yes**		**21**	**24**
**Renal/ urinary tract outcomes (CTCAE)**	**Yes**		**3 (3 at risk)**	**8 (7 at risk)**
Chronic kidney disease		1	3	7
		2	0	1
**Reproductive/ Genital**				
**Survivors at risk**	**Yes**		**23**	**23**
**Reproductive/ genital outcomes (CTCAE)**	**Yes**		**0**	**8 (8 at risk)**
Abnormal sperm concentration		2	0	1
Dyspareunia		2	0	1
Central hypogonadism		2	0	3
Precocious puberty		2	0	1
Primary ovarian failure		**3**	**0**	**1**
**Endocrine**				
**Survivors at risk**	**Yes**		**8**	**12**
**Endocrine CTCAE**	**Yes**		**13**	**16**
Abnormal glucose metabolism		**3**	**0**	**1**

Adrenal insufficiency		2	0	2
Adult growth hormone deficiency		1	0	4
Diabetes insipidus		2	0	3
Hyperthyroidism		2	1	0
Hypothyroidism		2	3	6
Obesity		2	7	9
		**3**	**2**	**4**
		**4**	**1**	**0**
Under weight		2	1	0
**Cardiovascular**				
**Survivors at risk**	**Yes**		**24**	**17**
**Cardiovascular CTCAE**	**Yes**		**14**	**14**
Heart valve disorder		2	0	2
High total cholesterol		1	2	4
Hypertension adult		1	10	11
		2	2	0
Hypertension pediatric		1	1	0
Hypertriglyceridemia		1	2	3
		2	1	0
		**3**	**0**	**1**
